# Seasonal dynamics and nutritional risk factors of gastric ulcers in fattening pigs: Results from a one-year field study in Slovakia

**DOI:** 10.17221/44/2025-VETMED

**Published:** 2025-11-27

**Authors:** Zuzana Krepelkova, Katarina Bardova, Frantisek Zigo, Arpad Csorgo, Jaroslav Novotny

**Affiliations:** ^1^Clinic of Swine, University of Veterinary Medicine and Pharmacy, Kosice, Slovak Republic; ^2^Department of Animal Nutrition and Husbandry, University of Veterinary Medicine and Pharmacy, Kosice, Slovak Republic; ^3^Private Veterinarian, ARTVET, Gabcikovo, Slovak Republic

**Keywords:** gastric ulcers, prevalence, seasonal variability, stomach content, swine

## Abstract

This study aimed to comprehensively evaluate the prevalence, severity, and risk factors associated with gastric lesions in fattening pigs across all four seasons in Slovakia. A total of 1 944 porcine stomachs were examined post-mortem at commercial slaughterhouses, focusing on the non-glandular region (*pars oesophagea*). A macroscopic evaluation was conducted using a standardised scoring system (0–3), in which gastric lesions, including parakeratosis, erosions, and ulcerations, were observed in 48% of the examined stomachs. Significant seasonal variation was detected, with the highest lesion prevalence recorded during the summer months, likely due to heat stress and reduced feed intake, and the lowest incidence of pathological changes seen in autumn. The gastric fullness had a notable impact: empty and liquid-filled stomachs were more frequently associated with severe mucosal damage, while full stomachs exhibited a protective effect. Furthermore, the feeding regimen played a crucial role: the pigs receiving wet feed had a significantly lower prevalence of gastric lesions than those on a dry feeding regimen. These results underscore the multifactorial nature of gastric ulceration in pigs and highlight the importance of nutritional and environmental management strategies in intensive production systems.

Gastric ulcers in pigs represent a significant health and economic concern with a global prevalence, particularly in intensive production systems for fattening pigs. These ulcerative lesions most commonly affect the non-glandular part of the stomach, the *pars oesophagea*, which is anatomically and functionally the most vulnerable region due to its thin, stratified squamous epithelium lacking natural protection against acidic gastric contents. The clinical consequences of such lesions range from parakeratosis and superficial erosions to deep ulcerations that may lead to reduced growth performance, anaemia, gastric perforation, peracute mortality, and contamination of carcasses with pathogens such as *Helicobacter suis* and *Fusobacterium gastrosuis* (Friendship et al. 2000; Canibe et al. 2016; Cybulski et al. 2024).

The pathogenesis of gastric ulcers in pigs is multifactorial, involving complex interactions among mechanical, nutritional, microbial, environmental, and stress-related factors. According to the literature, finely ground or pelleted feed is among the major risk factors, as it results in less voluminous gastric content and diminished mucosal protection (Eisemann and Argenzio 1999; Grosse Liesner et al. 2009; Cappai et al. 2013). The feed particle size has also been identified as a key nutritional factor by several authors, with finer particles (<400 μm) associated with a higher risk of mucosal damage, and coarser particles (600–700 μm) potentially offering a protective effect (Mikkelsen et al. 2004; Bao et al. 2016).

Additionally, a low dietary fibre intake leads to reduced coverage of the mucosa with mucus (Priester et al. 2020; Wang et al. 2020), while stressful environmental conditions such as elevated ambient temperature, ammonia, and dust can disrupt physiological homeostasis (Ramis 2006; Zhang et al. 2020). The type of gastric contents is also critical: the absence of solid ingesta or the presence of liquid content may fail to effectively buffer the acidic environment (Madec et al. 1998; Wilson et al. 1998). Some studies have also suggested a seasonal pattern in the occurrence of gastric ulcers, with a higher prevalence reported during the summer months, which may be attributed to reduced feed intake, dehydration, and increased heat stress (Friendship et al. 2000; Zhang et al. 2020). Among nutritional interventions, wet feed and including dietary fibre or coarse feed components have been shown to exert protective effects by slowing gastric emptying and improving the intragastric pH buffering capacity (Millet et al. 2012; Spanghero et al. 2024).

## MATERIAL AND METHODS

This prospective macroscopic study was conducted across all four seasons from March 2024 to March 2025, to analyse the prevalence, seasonal variability, and associated risk factors of ulcerative lesions in the *pars oesophagea* region of the stomach in fattening pigs in Slovakia. A total of 1 944 stomachs from pigs slaughtered at three commercial abattoirs located in western and eastern regions of Slovakia were examined. The animals originated from 11 commercial farms in the Trnava, Nitra, Prešov, and Košice regions. The herd composition included Yorkshire × Landrace × Duroc crossbreeds, with some farms also supplying Mangalica and Large White pigs. During the monitoring period, the farms maintained fattening pig populations ranging from approximately 1 000 animals on smaller farms to large operations with up to 40 000 pigs.

### Macroscopic evaluation

**Figure 1:**
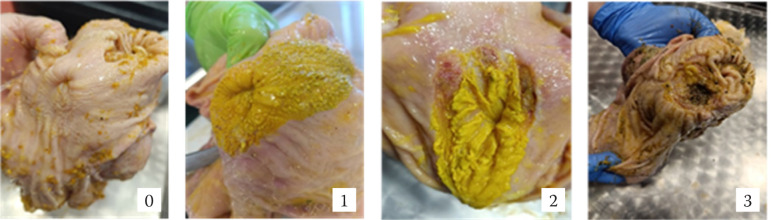
Macroscopic lesions in the *pars oesophagea* (score 0–3) Source: Authors’ own photographs

All stomachs were immediately opened and emptied post-mortem and evaluated macroscopically by a qualified examiner to minimise subjective variability. The mucosal surface of the *pars oesophagea* was assessed using a modified scoring system based on Robertson et al. (2002), ranging from 0 to 3: 0 = normal mucosa without visible lesions; 1 = parakeratosis; 2 = erosion; 3 = ulceration (gastric ulcer; Figure 1).

### Seasonal analysis

To assess seasonal variation, samples were collected and analysed during all four seasons:

– Spring (average seasonal temperature: 11.1 °C): 225 stomachs;
– Summer (average seasonal temperature: 21.8 °C): 257 stomachs;
– Autumn (average seasonal temperature: 10.9 °C): 615 stomachs;
– Winter (average seasonal temperature: –0.4 °C): 847 stomachs.

The data were processed and compared to identify periods with increased prevalence of lesions and to evaluate the potential influence of seasonal environmental factors.

### Effect of stomach contents

The state of gastric contents was also recorded (762 stomachs examined) during the examination and classified into three categories:

– Empty: 400 stomachs;
– Liquid content: 212 stomachs;
– Solid/full content: 150 stomachs.

For each category, the frequency of lesion scores (0–3) was recorded to analyse the impact of stomach fullness status on the occurrence and severity of ulcerations.

### Comparison of feeding regimens

The animals were divided into two fattening groups based on the type of diet they consumed:

– Dry feed: a standard commercial feed mixture for fattening pigs (OŠ-06, Table 1).
– Wet feed: the same feed mixture diluted with whey and an organic acid solution (*Schaumacid Drink S.*, Schaumann).

**Table 1:** Composition of the OŠ-06 feed mixture

Component/parameter	Typical value
Wheat meal	main cereal source
Maize (corn)	energy source
Barley meal	cereal improving digestibility
Soybean meal (extracted)	main protein source (usually non-GMO)
Vitamin–mineral premix	provides essential trace elements (Zn, Fe, Cu, Mn, Se, I) and vitamins (A, D_3_, E, B-complex)
Crude protein (CP)	approx. 13–13.2% (~132 g/kg)
Crude fat	approx. 2.2% (~22 g/kg)
Crude fibre	approx. 4% (~40 g/kg)
Ash	approx. 4.7% (~47 g/kg)
Calcium (Ca)	approx. 0.65%
Phosphorus (P)	approx. 0.55%
Sodium (Na)	approx. 0.18%
Lysine	approx. 0.9% (~9 g/kg)
Methionine	approx. 0.4% (~4 g/kg)

The stomach contents of 178 pigs in the dry-fed group and 214 pigs in the wet-fed group were macroscopically evaluated.

### Statistical analysis

To compare the prevalence of lesions between the groups (in terms of seasonal periods, gastric content, and feeding regimens), the chi-square test of independence (χ^2^ test) was used. A *P*-value < 0.05 was considered statistically significant.

## RESULTS

### Overall lesion prevalence in the *pars oesophagea*

**Figure 2:**
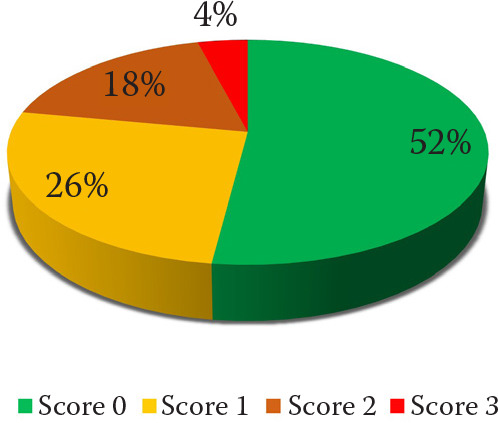
Annual incidence of gastric ulcers in fattening pigs from Slovakia

Out of 1 944 stomachs examined from the fattening pigs, a healthy mucosa (score 0) was observed in 52% of the animals. Pre-ulcerative lesions were present in 48% of the cases, including parakeratosis (score 1) in 26%, erosions (score 2) in 18%, and ulcerations (score 3) in 4% of the examined individuals (Figure 2). These findings confirm that gastric mucosal damage is a common subclinical condition in intensive pig production.

### Seasonal differences in lesion prevalence

**Figure 3:**
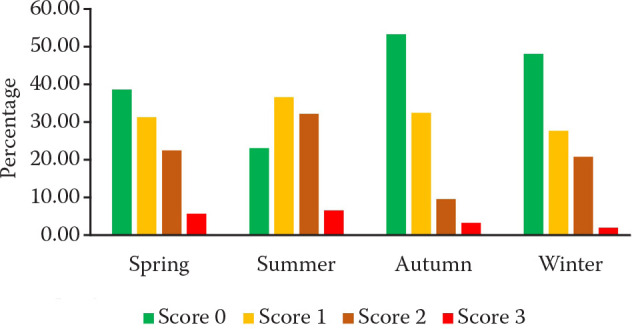
Seasonal occurrence of pars esophageal stomach lesions

The analysis by season revealed significant differences in the lesion frequency:

The highest proportion of healthy mucosa was observed in autumn (53.7%), followed by winter (48.5%) and spring (39%), and the lowest occurrence of healthy stomachs was found in summer (23.5%; Figure 3).

– Parakeratosis (score 1) was most prevalent in summer (37.05%) and least prevalent in winter (28.1%).
– Erosions (score 2) were also most frequent in summer (32.58%), while in other seasons, their occurrence ranged between 9.9% and 22.86%.
– Gastric ulcers (score 3) reached their highest incidence in summer (6.87%), followed by spring (6%), autumn (3.6%), and winter (2.25%).

These results suggest that the summer months represent the period with the highest risk for lesion development, likely due to heat stress.

### Effect of stomach contents on lesion prevalence

**Figure 4:**
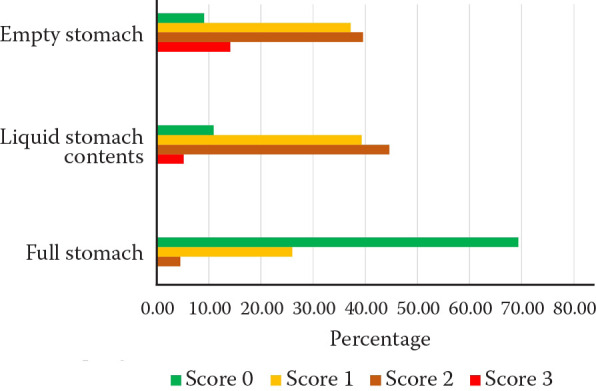
*Pars oesophagea* damage score depending on gastric fullness

Out of 762 stomachs evaluated, significant differences in mucosal condition were observed depending on the stomach contents:

– Empty stomachs: Only 9.1% of these stomachs had healthy mucosa, and parakeratosis was present in 37.2%, erosions in 39.6%, and ulcerations in 14.1% (Figure 4).
– Liquid content**:** healthy mucosa was found in 10.9%**,** parakeratosis in 39.3%**,** erosions in 44.65%**,** and ulcerations in 5.15% of these stomachs**.**
– Full stomachs**:** healthy mucosa was observed in 69.38%**,** parakeratosis in 26%**,** erosions in 4.5%**,** and ulcerations in only 0.12% of these stomachs**.**

A statistical analysis using the chi-square test of independence revealed a highly significant difference between the groups (χ^2^ ≈ 43.6; *P* < 1.04 × 10^–12^), confirming that a full stomach exerts a protective effect on the *pars oesophagea* and significantly reduces the risk of ulcerations.

### Effect of feed type

**Figure 5:**
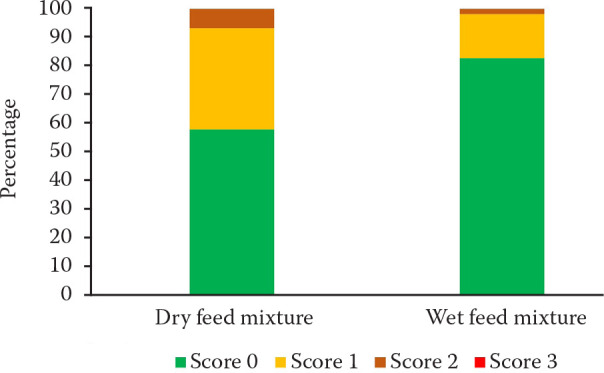
Feeding type vs lesion incidence

For the 392 stomachs evaluated (178 dry-fed, 214 wet-fed), the following results were recorded (Figure 5):

– Dry-fed: score 0–103 stomachs; score 1–63; score 2–12; score 3–0.
– Wet-fed: score 0–177 stomachs; score 1–33; score 2–4; score 3–0.

Wet feed was associated with a higher proportion of healthy mucosa (82.7%) and a lower prevalence of parakeratosis and erosions than that of dry feed (57.9% healthy). The results of the chi-square test of independence confirmed the statistical significance of differences between the groups (χ^2^ = 29.88; *P* = 3.25 × 10^–7^), confirming that the type of feed significantly affects the occurrence of mucosal damage in the *pars oesophagea*.

## DISCUSSION

Gastroesophageal ulcers represent a significant health and economic issue in pig fattening and are increasingly recognised as an essential welfare concern in both fattening pigs and culled sows (Cybulski et al. 2021). Our study focused on the occurrence and severity of lesions in the *pars oesophagea* region, identifying multiple factors contributing to their development. The findings clearly indicate that the aetiology of these ulcers is multifactorial, involving stomach anatomy, seasonal conditions, digestive tract fullness, and the type of feed administered.

Additionally, alterations in the gastric microbiome have been linked to the development of ulcerative lesions, highlighting the importance of microbial factors alongside nutritional and mechanical influences (De Rodas et al. 2018).

Lesions localised in the *pars oesophagea*, the nonglandular part of the stomach, develop progressively, starting as parakeratosis and hyperkeratosis that worsen to erosions and deep ulcers, which may lead to acute haemorrhage or even stenosis of the distal oesophagus (Friendship 2022). The underlying cause is the absence of a mucosal barrier typical for the glandular stomach, making the *pars oesophagea* particularly susceptible to chemical and mechanical injury. In our study, we applied Robertson’s classification (Robertson et al. 2002), an established standardised tool for assessing macroscopic changes in the gastric mucosa.

From the macroscopic analysis of 1 944 pig stomachs, up to 48% showed pre-ulcerative or ulcerative changes (score three lesions in 4% of the pigs), indicating a high disease prevalence. These values align with abattoir findings reported by Swaby and Gregory (2012) and with Mushonga et al. (2017), who reported ulcerations in 5.1% of animals in South Africa, and with more recent data from Lin et al. (2024), where the prevalence reached 15%, possibly reflecting deteriorating conditions in intensive farming systems.

A key finding of our study is the marked seasonality of lesion occurrence. The highest incidence of ulcers was observed during the summer months, consistent with the conclusions of Hessing et al. (1994) and Ramis (2006). Gottardo et al. (2017) identified heat stress as a primary trigger for gastric mucosal damage. Although the average temperature in Slovakia during the study period was approximately 21.8 °C, the highest recorded air temperature reached 38.3 °C. High temperatures lead to reduced feed and water intake, increased acid secretion, and weakening of the mucosal barrier (Liu et al. 2019; Ortega and Szabo 2021). Zhang et al. (2020) report that gastric hypomotility and increased mucosal exposure to acidic contents promote ulcer formation during hot days. Conversely, the pig stomachs examined during autumn showed the highest proportion of healthy mucosa, likely due to milder temperatures, lower stress, and more stable feed intake (Amory et al. 2002; Albert et al. 2024). Tang et al. (2022) further emphasised the importance of environmental stress in gastric ulcer pathogenesis. From a preventive standpoint, it is crucial to tailor preventive measures to seasonal fluctuations, for example, focusing on reducing heat stress, optimising ventilation, and supporting feed intake during summer (Gottardo et al. 2017; Zhang et al. 2020).

The stomach fullness was found to be a significant factor, with the lowest ulcer prevalence observed in animals with a full stomach, supporting the theory that a full stomach acts as a physical barrier protecting the *pars oesophagea* from acid and mechanical irritation (Wilson et al. 1998; Herskin et al. 2016). Conversely, an empty stomach was clearly associated with the worst lesion scores, consistent with findings from Zimmerman et al. (2012) and Doster (2000). Prolonged fasting leads to mucosal overexposure to acidic conditions, significantly contributing to ulcer pathogenesis (Cybulski et al. 2024). An interesting intermediate was animals with liquid stomach contents, which provided only partial protection, indicating that mechanical protection is as important as chemical protection, as Madec et al. (1998) and Grosse Liesner et al. (2014) noted. Similar findings were reported by Peralvo-Vidal et al. (2021), who demonstrated a significant association between increased gastric content fluidity and a higher prevalence of *pars oesophagea* ulcers in nursery pigs from high-risk commercial herds. Notably, not only the quantity but also the consistency of the feed plays a crucial role; hence, pre-slaughter feeding management is important. Efficient emptying of the digestive tract reduces the risk of faecal contamination of carcasses (Warriss 2003), but fasting longer than 24 h increases the ulcer risk (Madec et al. 1999). EFSA (2011) recommends a fasting interval of 12–18 h to allow for safe processing without compromising animal welfare. This compromise is also economically crucial for reducing penalties for contamination and lowering the gastrointestinal content weight (Velarde et al. 2000), but it must not endanger animal health. In our annual survey, we identified three main stomach fullness states at slaughter: empty stomachs (52.5%), stomachs containing liquid content (27.8%), and stomachs filled with feed (19.7%).

The feed type was found to be another important factor in ulcer development. Our study supports the hypothesis that wet feed protects against ulcer development. The softer feed consistency reduces mechanical mucosal damage, accelerates gastric passage, and prevents particle segregation that could create regions with varying pH (De Groote et al. 1996; Grosse Liesner et al. 2009). Increased salivation during wet feeding also enhances the buffering capacity of gastric contents, further reducing the erosion risk (Da Silva et al. 2013). Gresse et al. (2017) added that wet feed may positively influence the gut microbiota, thereby secondarily supporting mucosal health. Moreover, the feed type and particle size significantly affect pigs’ growth performance, nutrient digestibility, and gastric health (Jo et al. 2021). The ulcer prevalence is higher when diets contain a large proportion of very fine particles, which can lead to rapid gastric emptying and prolonged exposure of the *pars oesophagea* to acidic content (Wondra et al. 1995; Dritz et al. 2002). The proportion of particles <0.4 mm is particularly critical, with values exceeding 30% substantially increasing the lesion risk (Grosse Liesner et al. 2014). Coarser particle sizes (around 600–700 μm) help maintain a more stable gastric environment and are associated with lower ulcer prevalence, a finding consistent with our observation that diets providing more structural content tend to be less harmful. Therefore, future preventive strategies should address the feed composition and its physical characteristics.

This study provides a comprehensive and up-to-date assessment of the prevalence of gastric ulcers in fattening pigs in Slovakia. Based on abattoir data, 48% of the examined stomachs displayed visible pre-ulcerative lesions, including parakeratosis (26%), erosions (18%), and gastric ulcers (4%). These findings highlight the high occurrence of subclinical gastric lesions in fattening pigs and emphasise the importance of systematic monitoring and identification of aetiological factors to improve pig health and welfare. Our results confirmed a significant seasonal influence of environmental factors on gastric ulcer development, with summer being the most critical period. Increased heat stress, reduced feed intake, and overall physiological burden during hot months compromise the mucosal defence mechanisms, leading to a higher risk of ulceration. In contrast, autumn was associated with more favourable conditions and a lower incidence of mucosal damage. These results underline the need for targeted prevention strategies to mitigate environmental stressors, particularly in summer. This involves ensuring adequate access to cool drinking water, shade, effective ventilation, and optimised nutrition. Furthermore, our findings clearly demonstrated the importance of the stomach contents in protecting the *pars oesophagea*: the lowest lesion prevalence was observed in pigs with full stomachs, where the feed mass likely acted as a physical barrier against gastric acid. Liquid contents provided only partial protection, whereas empty stomachs were most frequently associated with ulceration. These findings point to the necessity of regular feeding and proper fasting management before slaughter to minimise mucosal damage without compromising carcass hygiene and quality. Lastly, the type of feed was shown to affect lesion occurrence significantly: wet feed was associated with a higher proportion of healthy mucosa and a lower prevalence of parakeratosis and erosions. The beneficial effect of wet feed is likely due to mechanical, chemical, and microbiological factors such as reduced abrasive irritation, increased salivation, and improved microbial balance in the stomach. Overall, this study contributes to a better understanding of the multifactorial aetiology of gastric ulcers and highlights several practical opportunities for their prevention. Effective herd management, tailored nutrition strategies, and consideration of seasonal influences represent key factors in reducing ulcer prevalence and enhancing health, welfare, and performance in fattening pigs.
